# Is Huachansu Beneficial in Treating Advanced Non-Small-Cell Lung Cancer? Evidence from a Meta-Analysis of Its Efficacy Combined with Chemotherapy

**DOI:** 10.1155/2015/408145

**Published:** 2015-08-11

**Authors:** Bingduo Zhou, Fengying Wu, Lin Yuan, Zhulei Miao, Shengliang Zhu

**Affiliations:** ^1^Department of Gastroenterology, Yueyang Hospital of Integrated Traditional Chinese and Western Medicine, Shanghai University of Traditional Chinese Medicine, Shanghai 200437, China; ^2^Department of Oncology, Shanghai Pulmonary Hospital, Tongji University School of Medicine, Shanghai 200433, China; ^3^Science and Technology Experimental Center, Shanghai University of Traditional Chinese Medicine, Shanghai 201203, China; ^4^Department of Immunology and Pathogen Biology, School of Basic Medicine, Shanghai University of Traditional Chinese Medicine, Shanghai 201203, China

## Abstract

*Background*. Huachansu, the sterilized water extract of *Bufo bufo gargarizans* toad skin, is used in China to alleviate the side-effects and enhance the therapeutic effect of chemotherapy in advanced non-small-cell lung cancer (NSCLC). We conducted a meta-analysis to assess Huachansu's efficacy. *Methods*. We extensively searched electronic databases (CENTRAL, EMBASE, MEDLINE, CBM, Cochrane Library, CNKI, CEBM, WFDP, CSCD, CSTD, and IPA) for randomized controlled trials containing Huachansu plus chemotherapy as the test group and chemotherapy as the control group. Seventeen trials were selected based on the selection criteria. The pooled relative ratio (RR) of indicators with 95% confidence interval (95% CI) was calculated for efficacy evaluation. *Results*. The meta-analysis demonstrated a statistically significant improvement in objective tumor response, one-year survival, Karnofsky performance status, pain relief, and alleviation of severe side-effects (nausea and vomiting, leukocytopenia) in the test group as compared to the control group, but no significant difference in thrombocytopenia. *Conclusions*. This study demonstrated the efficacy of Huachansu combined with chemotherapy in the treatment of advanced NSCLC. However, limitations exist and high-quality trials are needed for further verification.

## 1. Introduction

Non-small-cell lung cancer (NSCLC) is the most common type of lung cancer, which is difficult to diagnose in early stages. Patients diagnosed with advanced NSCLC are often unable to undergo surgery, thus reducing the survival time [1]. So far, chemotherapy is typically used for the treatment of advanced NSCLC. However, chemotherapy not only kills tumor cells but also causes several side-effects. Many patients have to endure great discomfort to prolong their lives for a limited time. So providing efficacious therapy while improving the patient's quality of life is the primary concern of clinicians when selecting appropriate regimens.

Huachansu (Cinobufacini) is the sterilized water extract of* Bufo bufo gargarizans* toad skin [2], a small amphibian animal that is widely distributed in China and has survived on the earth for millions of years. During this time, the toad's skin developed the ability to produce various active ingredients to protect itself from adverse environmental factors [3]. These ingredients include alkaloids, peptides, and steroids, which inhibit the growth of fungi, bacteria, and viruses, stimulate nerves, and induce anesthesia (a powerful weapon against predators) [4]. Moreover, other ingredients have been identified, which kill cancer cells, induce apoptosis, prevent angiogenesis, and enhance the immune system [5, 6], which make the skin of* Bufo bufo gargarizans* a valuable source of traditional Chinese medicine (TCM) [7, 8]. The Chinese Food and Drug Administration approved Huachansu in 1991 for the treatment of chronic HBV infection and cancers (predominantly as standard-of-care monotherapy for pancreatic and hepatobiliary malignancies) [9].

In order to alleviate the side-effects of chemotherapy and enhance therapeutic efficacy in advanced NSCLC, several clinical studies have been conducted using Huachansu combined with chemotherapy to observe whether this would be beneficial for patients. But due to the limited scale of these studies, certain results remain inconsistent. Therefore, we performed a meta-analysis by pooling the randomized clinical trials to evaluate the efficacy of this complementary therapy.

## 2. Material and Methods

### 2.1. Search Strategy

Clinical trials were retrieved from the Cochrane Central Register of Controlled Trials (CENTRAL); EMBASE; MEDLINE; Cochrane Library; Chinese Biological Medicine Database (CBM); China National Knowledge Infra-Structure Database (CNKI); Chinese Evidence-Based Medicine Database (CEBM); Wan Fang Digital Periodicals Database (WFDP); China Science Citation Database (CSCD); Chinese Science Technology Document Database (CSTD); and the International Pharmaceutical Abstracts (IPA), from their inception to January 2015. The retrieval terms used were as follows: (non-small-cell lung cancer OR non-small-cell lung carcinoma OR NSCLC OR squamous cell lung carcinoma OR large cell lung carcinoma OR lung adenocarcinoma) AND (Huachansu OR Hua chan su OR Cinobufacini).

### 2.2. Study Selection

Studies that met the following criteria were included: (1) type of studies: clinical randomized controlled trials (RCTs); (2) participants: age ≥18 years with pathology and CT diagnosis of NSCLC in stage III or IV with detectable solid tumor and KPS ≥60; (3) type of intervention: treatment with Huachansu combined with chemotherapy as the test group; (4) type of outcome: reports with at least one of the following indicators: objective tumor response, one-year survival, quality of life (KPS scale), pain relief, major drug adverse reactions, or the data necessary to calculate them. Clinical trials were excluded if they did not meet the above criteria. The following types of studies were also excluded: (1) treatment without chemotherapy as the control group; (2) nonoriginal or duplicated publications; (3) patients with other serious illnesses.

### 2.3. Quality Assessment and Data Extraction

All reports were independently reviewed by two reviewers (BD Zhou and FY Wu) to evaluate their quality, decide which study would meet the eligibility criteria, and extract the data needed: author names, year of publication, study type, patient information, details of the treatment, and outcome. Any disagreements were resolved through discussion or by a third reviewer.

### 2.4. Meta-Analysis

The STATA (version 11.0) was used for data analysis. Relative ratio (RR) and 95% confidence intervals (CI) were calculated. *p* < 0.05 was considered to be statistically significant. To determine whether the random-effects or fixed-effects model should be used, the *I* ^2^ was used to estimate heterogeneity (random-effects model when *I* ^2^ ≥ 50% or fixed-effects model when *I* ^2^ < 50%) [27].

The indicators for evaluation of therapeutic efficacy were as follows: (1) objective tumor response; (2) one-year survival; (3) improved performance status; (4) pain relief; (5) symptoms of severe side-effects caused by chemotherapy (nausea and vomiting, leukocytopenia, and thrombocytopenia).

## 3. Results

### 3.1. Description of Enrolled Studies

**Figure 1:**
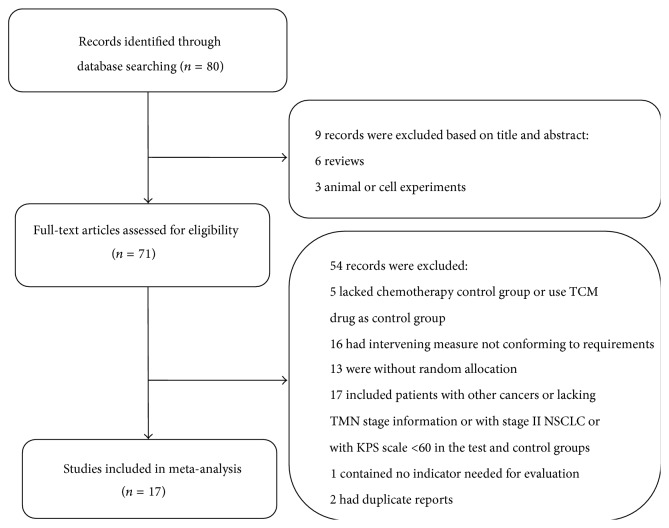
Flow chart of study selection.

In this study, seventeen trials were selected based on selection criteria. A total of 80 publications were originally identified, of which 63 studies were excluded and 17 studies were finally included based on the selection criteria with 1142 enrolled patients (578 in the test group and 564 in the control group) [10–26]. The selection process and reasons for exclusion are described in Figure 1, and the characteristics of the included studies are presented in Table 1.

All 17 studies mentioned random allocation, but only six studies described random allocation methods. No study described the blind method and allocation concealment. All patients recruited in these studies were diagnosed at stages III to IV NSCLC TNM. These patients were given an overall physical assessment before treatment, including age, quality of life score, and stage of disease. No significant differences between the baseline data were reported and all patients were estimated with life expectancy ≥3 months. Different combination chemotherapies were used for treatment: vinorelbine plus cisplatin (NP), docetaxel plus cisplatin (DC), paclitaxel plus cisplatin (TP), gemcitabine plus cisplatin (GP), etoposide plus cisplatin (EP), vinorelbine plus carboplatin (NC), vinorelbine plus ifosfamide (NI), and pemetrexed plus cisplatin. The methodological quality was evaluated using the Jadad scale according to the description in these reports on randomization, blinding method, withdrawals/dropouts, and allocation concealment, which was presented in Table 2.

### 3.2. Objective Tumor Response Results

**Figure 2:**
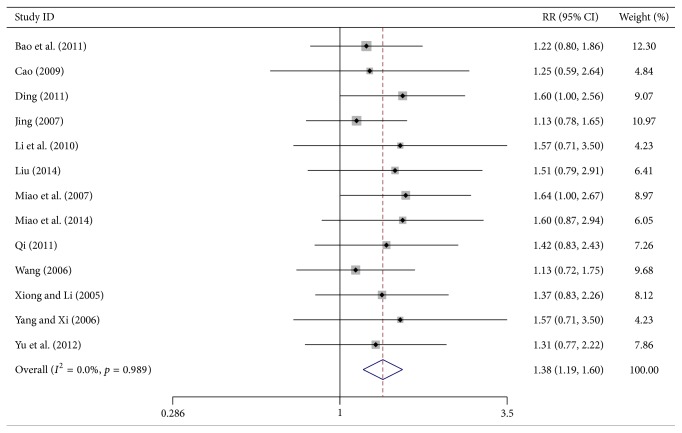
Forest-plot of objective tumor response.

Thirteen trials reported the number of treated patients with complete response (CR), partial response (PR), stable disease (SD), and progressive disease (PD) in each group, based on the WHO scale. The objective tumor response was determined by the number of patients with CR plus PR. Meta-analysis showed a significant increase in the number of patients with CR plus PR in the Huachansu plus chemotherapy test group (RR = 1.379, 95% CI, 1.190–1.599, *p* < 0.0001, 879 patients). A fixed-effects model was used since heterogeneity was absent (*I* ^2^ = 0%, *p* = 0.989) (Figure 2).

### 3.3. One-Year Survival Results

**Figure 3:**
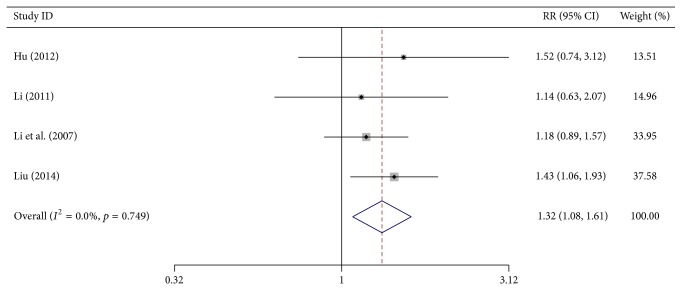
Forest-plot of one-year survival.

Four trials reported the number of treated patients who survived >1 year in each group. Meta-analysis showed a significant increase in the number of patients surviving > one year in the test group as compared to the control group (RR = 1.316, 95% CI, 1.077–1.607, *p* = 0.007, 262 patients). As heterogeneity was absent (*I* ^2^ = 0%, *p* = 0.749), a fixed-effects model was used (Figure 3).

### 3.4. Performance Status Results

**Figure 4:**
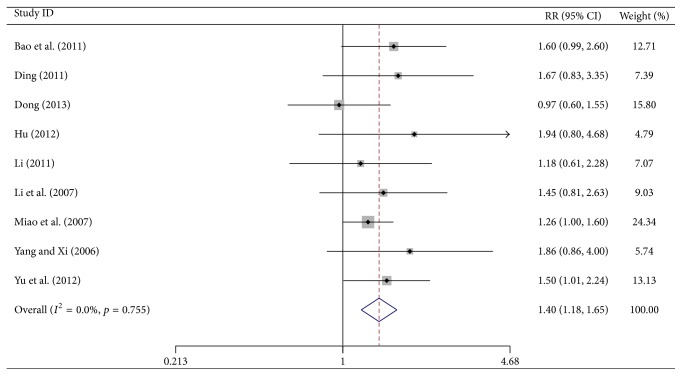
Forest-plot of improved Karnofsky performance status.

Nine trials reported the number of patients with improved status (an increase of ≥10 points), stable status (an increase or decrease of <10 points), and decline status (a decrease of ≥10 points) in each group based on the Karnofsky performance scale (KPS). Meta-analysis showed a significant increase in the number of patients with improved status in the test group (RR = 1.397, 95% CI, 1.185–1.648, *p* < 0.0001, 645 patients). Since heterogeneity was absent (*I* ^2^ = 0%, *p* = 0.755), a fixed-effects model was used (Figure 4).

### 3.5. Pain Relieving Effects

**Figure 5:**
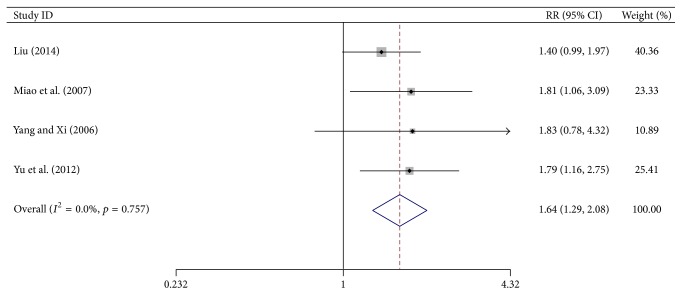
Forest-plot of pain relief.

Four trials reported the number of patients with pain relief after treatment. The degree of pain was assessed with the WHO rating scale. Meta-analysis showed a significant increase in the number of patients with complete plus partial pain relief in the test group (RR = 1.64, 95% CI, 1.293–2.080, *p* < 0.0001, 296 patients). Since heterogeneity was absent (*I* ^2^ = 0%, *p* = 0.757), a fixed-effects model was used (Figure 5).

### 3.6. Severe Chemotherapy Toxicity Results

**Figure 6:**
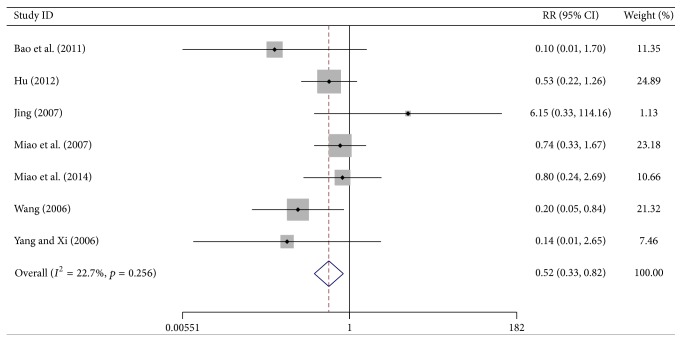
Forest-plot of nausea and vomiting at the grade of III~IV.

**Figure 7:**
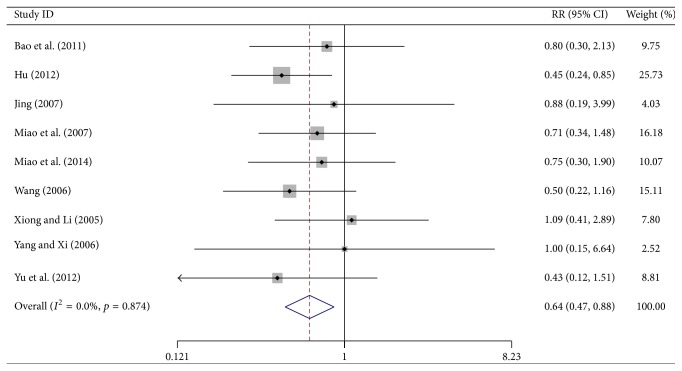
Forest-plot of leukocytopenia at the grade of III~IV.

**Figure 8:**
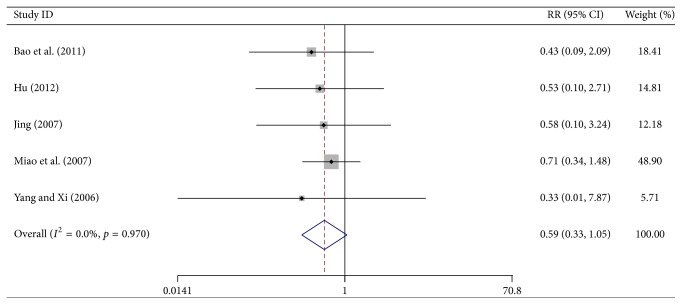
Forest-plot of thrombocytopenia at the grade of III~IV.

Severe chemotherapy toxicities such as gastrointestinal side-effects and myelosuppression are among the most important causes for patients' failure to treatment adherence. Seven trials reported the number of cases with nausea and vomiting of grade III or IV (WHO scale). Meta-analysis showed a significant reduction in the number of cases in the test group as compared to the control group (RR = 0.523, 95% CI, 0.333–0.822, *p* = 0.005, 494 patients). A fixed-effects model was used, as the heterogeneity was not significant (*I* ^2^ = 22.7%, *p* = 0.256) (Figure 6). Nine trials reported the number of patients with leukocytopenia of grade III or IV (WHO scale). Meta-analysis showed a significant decrease in the number of patients with grade III or IV leukocytopenia in the test group (RR = 0.644, 95% CI, 0.473–0.876, *p* = 0.005, 620 patients). Since no heterogeneity was observed (*I* ^2^ = 0%, *p* = 0.874), a fixed-effects model was used (Figure 7). Five trials reported the number of treated patients with thrombocytopenia of grade III or IV (WHO scale). Although a trend of improvement was observed in the test group, there was no statistically significant difference between the two groups (RR = 0.593, 95% CI, 0.334–1.054, *p* = 0.075, 374 patients). As heterogeneity was absent (*I* ^2^ = 0%, *p* = 0.970), a fixed-effects model was used (Figure 8).

### 3.7. Sensitivity Analysis Results

The fixed-effects and random-effects models were used to perform sensitivity analysis. Though the *p* value increased for certain indicators when using the random-effects model, the outcomes did not change. The results are displayed in Table 3.

### 3.8. Publication Bias Analysis

**Figure 9:**
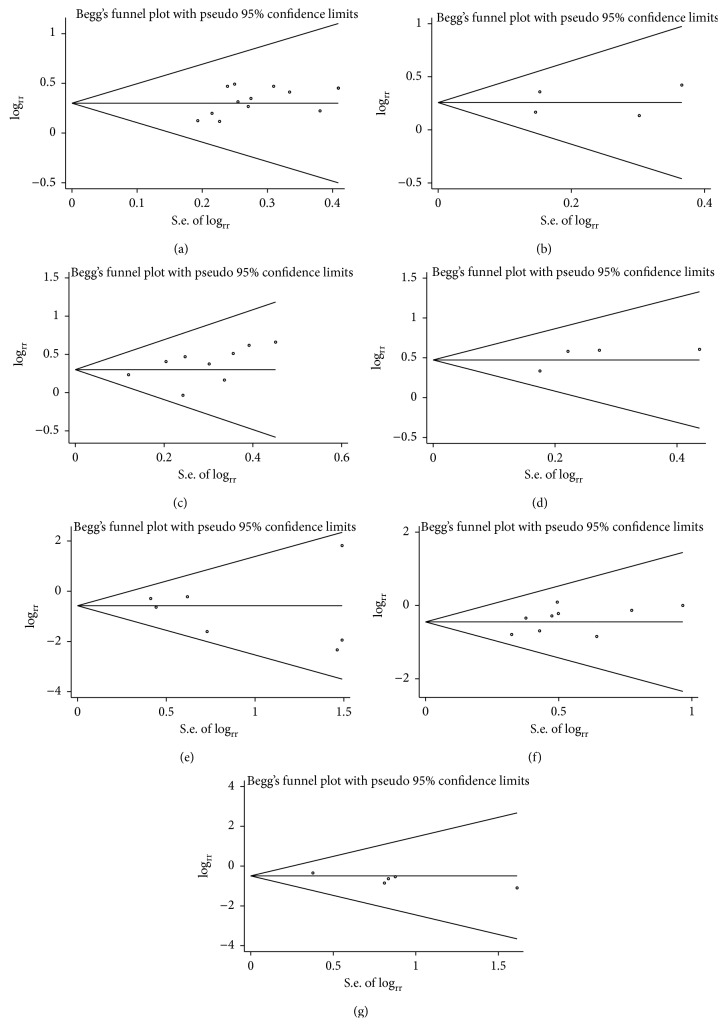
Funnel plots of the studies belong to different groups: (a) objective tumor response; (b) one-year survival; (c) improved Karnofsky performance status; (d) pain relief; (e) nausea and vomiting; (f) leukocytopenia; (g) thrombocytopenia.

Begg's funnel plot and Egger's linear regression test were used to evaluate the publication bias. The result of Egger's test suggested a publication bias among the studies within the group of thrombocytopenia (*p* = 0.036). No bias was observed in the other groups. The results are displayed in Figure 9 and Table 4.

## 4. Discussion

So far Huachansu has been used mainly in China, and most of the clinical trials were performed by separate hospitals with a small sample size. The number of patients included in most trials was < 100. So certain results were not very convincing. For example, five studies claimed no statistically significant improvement in the objective tumor response in the test group. A meta-analysis offered the unique advantage of merging all these outcomes to get a more comprehensive and accurate evaluation of such complementary therapy.

This meta-analysis showed that Huachansu can increase the objective tumor response and one-year survival, improve performance status, alleviate severe chemotherapy side-effects, and relieve cancer pain, which may be attributed to the various ingredients in Huachansu, which is just like a troop equipped with multiple weapons. So far, more than 30 ingredients have been identified in the skin secretion of* Bufo bufo gargarizans* and the major functional ingredients include cardiac glycosides (Cinobufagin, Resibufogenin, Bufalin, etc.) and indole alkaloids (Bufotenine, Cinobufotenine, Serotonin, etc.). Experiments have shown that Cinobufagin, Resibufogenin, and Bufalin strongly inhibit tumor cells, and Bufotenine, Cinobufotenine, and Serotonin can act on the neural system [5, 28]. Therefore, the clinical effects of Huachansu correspond well with the physiological activities of these ingredients.

## 5. Conclusions

In summary, this meta-analysis suggests that Huachansu, as a natural medicine containing many active ingredients, can be a promising supplement to routine chemotherapy in treating advanced NSCLC. However a major limitation of this study is the low methodological quality of the included reports. Therefore, more high-quality trials are needed in the future to validate these findings. Besides, to avoid publication bias, the likelihood of publication of trials should in no way depend on whether the results were positive or negative.

## Figures and Tables

**Table 1:** Characteristics of the included studies.

Study	Year	Stage of disease	Sample size (case/control)	Sex (F/M)	Age (median or mean or range)	Intervention		Treatment duration (week)	Description of random allocation method
						Test group	Control group		
Bao et al. [10]	2011	III, IV	45/48	N	56/52 (T/C)	Huachansu + GP	GP	6	N
Cao [11]	2009	III, IV	25/25	22/28	58	Huachansu + GP	GP	9	N
Ding [12]	2011	III, IV	39/39	14/64	53	Huachansu + NI	NI	6	N
Dong [13]	2013	IV	46/40	39/47	46~66/48~69 (T/C)	Huachansu + pemetrexed + DDP	Pemetrexed + DDP	12	Random number table
Hu [14]	2012	III, IV	36/38	31/43	36~66	Huachansu + TP	TP	12~18	N
Jing [15]	2007	III, IV	32/28	18/42	65/65 (T/C)	Huachansu + NC	NC	8	By the order of admission
Li [16]	2011	III, IV	21/18	14/25	57.5/61 (T/C)	Huachansu + DC	DC	9~12	N
Li et al. [17]	2007	III, IV	32/32	11/53	56.3	Huachansu + NP/GP	NP/GP	2	N
Li et al. [18]	2010	III, IV	30/30	22/38	54	Huachansu + NP/EP	NP/EP	6	N
Liu [19]	2014	III, IV	45/40	38/47	47.5 ± 11.33	Huachansu + NP/TP	NP/TP	6	Random number table
Miao et al. [20]	2007	III, IV	43/44	37/50	54 ± 20/53 ± 19 (T/C)	Huachansu + NP	NP	9~18	Envelope randomization
Miao et al. [21]	2014	III, IV	30/30	29/31	58.0 ± 7.0/57.0 ± 6.5 (T/C)	Huachansu + TP	TP	12	Random number table
Qi [22]	2011	III, IV	30/30	12/48	53	Huachansu + TP/GP/NP	TP/GP/NP	6	N
Wang [23]	2006	III, IV	30/30	18/42	58.8/60.2 (T/C)	Huachansu + TP	TP	4	N
Xiong and Li [24]	2005	III, IV	32/30	20/42	49.7	Huachansu + NP	NP	8	By the order of admission
Yang and Xi [25]	2006	III, IV	30/30	22/38	52	Huachansu + NP	NP	6	N
Yu et al. [26]	2012	III, IV	32/32	25/39	64/62 (T/C)	Huachansu + DC	DC	4	N

N: not mentioned; F: female; M: male; DDP = cisplatin; NP = vinorelbine + cisplatin; NC = vinorelbine + carboplatin; TP = paclitaxel + cisplatin; DC = docetaxel + cisplatin; GP = gemcitabine + cisplatin; EP = etoposide + cisplatin; NI = vinorelbine + ifosfamide; T/C: test group/control group.

**Table 2:** Methodological quality assessment using the Jadad scale.

Study	Randomization	Description of randomization methodology	Blinding	Description of blinding methodology	Description of withdrawals/dropouts	Allocation concealment	Jadad score
Bao et al. [10]	Yes	No	No	No	No	No	1
Cao [11]	Yes	No	No	No	No	No	1
Ding [12]	Yes	No	No	No	No	No	1
Dong [13]	Yes	Yes	No	No	No	No	2
Hu [14]	Yes	No	No	No	No	No	1
Jing [15]	Yes	Yes, but inappropriate	No	No	No	No	0
Li [16]	Yes	No	No	No	No	No	1
Li et al. [17]	Yes	No	No	No	No	No	1
Li et al. [18]	Yes	No	No	No	No	No	1
Liu [19]	Yes	Yes	No	No	No	No	2
Miao et al. [20]	Yes	Yes	No	No	No	No	2
Miao et al. [21]	Yes	Yes	No	No	No	No	2
Qi [22]	Yes	No	No	No	No	No	1
Wang [23]	Yes	No	No	No	No	No	1
Xiong and Li [24]	Yes	Yes, but inappropriate	No	No	No	No	0
Yang and Xi [25]	Yes	No	No	No	No	No	1
Yu et al. [26]	Yes	No	No	No	No	No	1

**Table 3:** Result of sensitivity analysis of the studies belonging to different indicator groups.

Indicator	Number of trials	Fixed-effects model			Random-effects model		
		RR	95% CI	*p* value	RR	95% CI	*p* value
Objective tumor response	13	1.379	1.190–1.599	*p* < 0.0001	1.351	1.168–1.562	*p* < 0.0001
One-year survival	4	1.316	1.077–1.607	*p* = 0.007	1.293	1.071–1.561	*p* = 0.008
Performance Status	9	1.397	1.185–1.648	*p* < 0.0001	1.351	1.160–1.572	*p* < 0.0001
Pain relief	4	1.640	1.293–2.080	*p* < 0.0001	1.604	1.273–2.022	*p* < 0.0001
Nausea and vomiting	7	0.523	0.333–0.822	*p* = 0.005	0.538	0.297–0.974	*p* = 0.041
Leukocytopenia	9	0.644	0.473–0.876	*p* = 0.005	0.638	0.468–0.871	*p* = 0.005
Thrombocytopenia	5	0.593	0.334–1.054	*p* = 0.075	0.611	0.344–1.083	*p* = 0.092

**Table 4:** Result of Begg's and Egger's test for publication bias of the studies belonging to different indicator groups.

Indicator	Number of trials	Publication bias	
		*p* value (Begg's)	*p* value (Egger's)
Objective tumor response	13	0.143	0.06
One-year survival	4	0.734	0.888
Performance status	9	0.076	0.19
Pain relief	4	1.000	0.300
Nausea and vomiting	7	0.764	0.603
Leukocytopenia	9	0.175	0.212
Thrombocytopenia	5	0.806	0.036
